# Supplementary figures and images for: Molecular insights into programmed cell death in esophageal squamous cell carcinoma
Source: PeerJ. 2024 Jul 10;12:e17690. doi: 10.7717/peerj.17690 (PMC11246021; doi:10.7717/peerj.17690)

**A**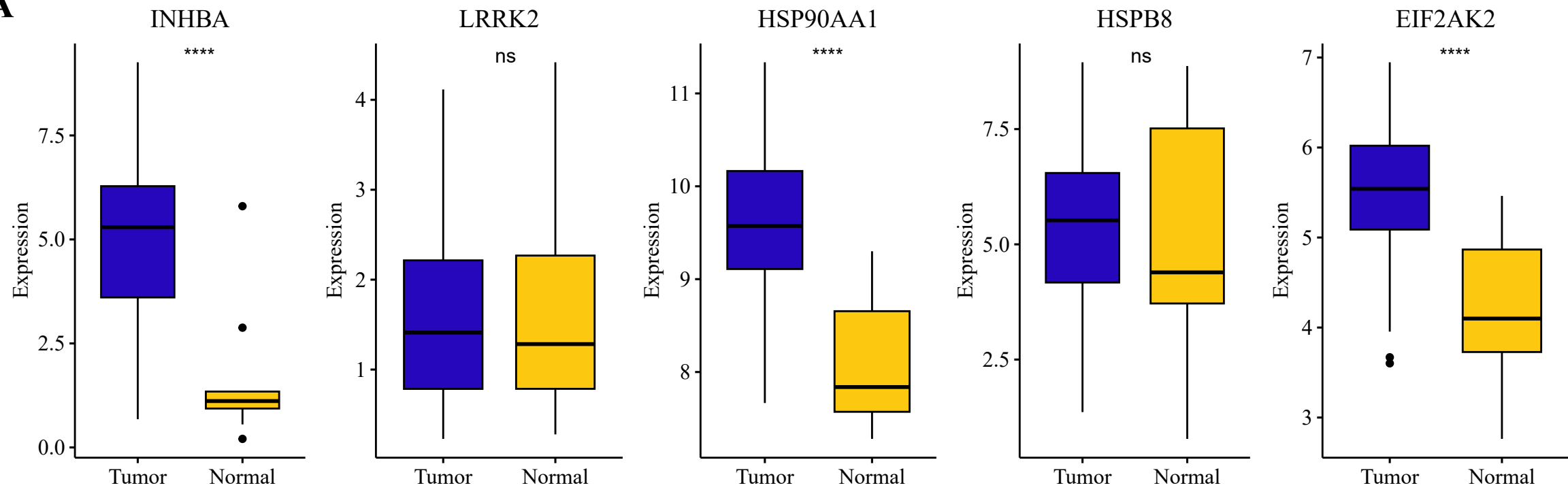**B**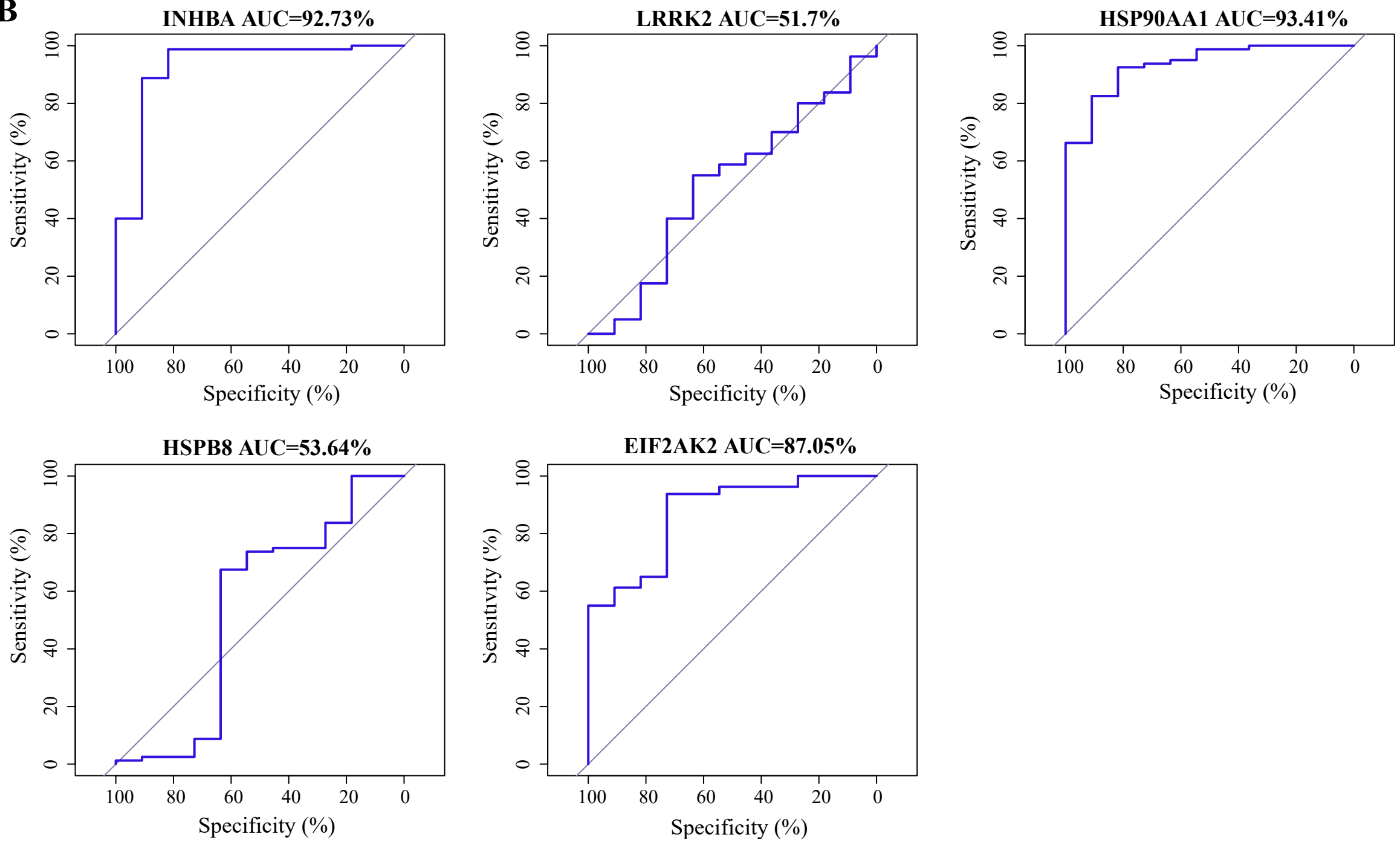

Supplement: Supplemental Information 2 — (A) Validation of 5 hub gene expression differences between tumor and normal samples. Wilcoxon rank sum test to compare differences between tumor and normal control samples. (B) ROC curves to validate 5 diagnostic markers. **** indicates p < 0.0001, and ns represents no significant difference. Data were presented as median. [file peerj-12-17690-s002.pdf]

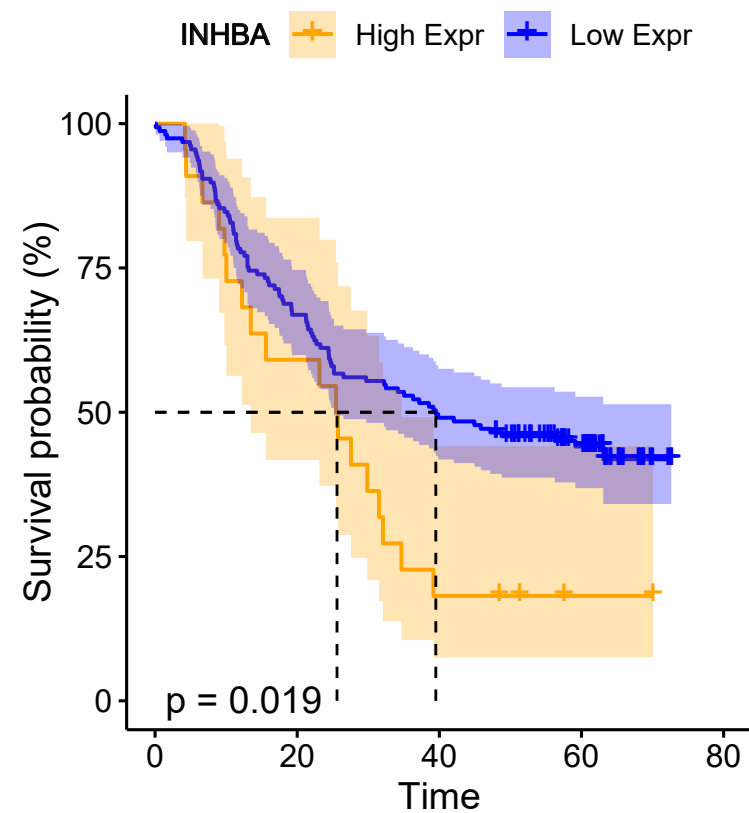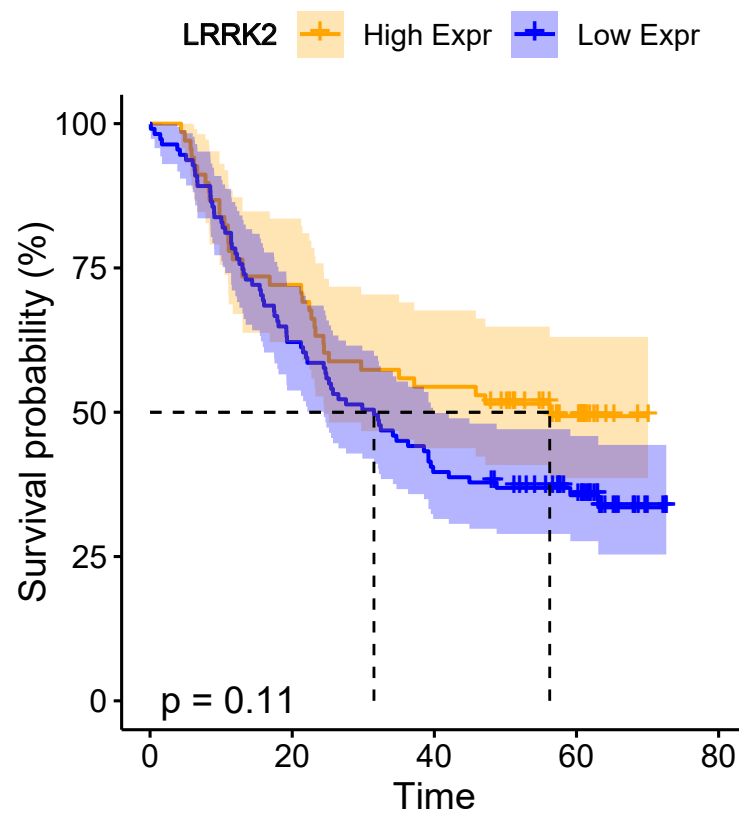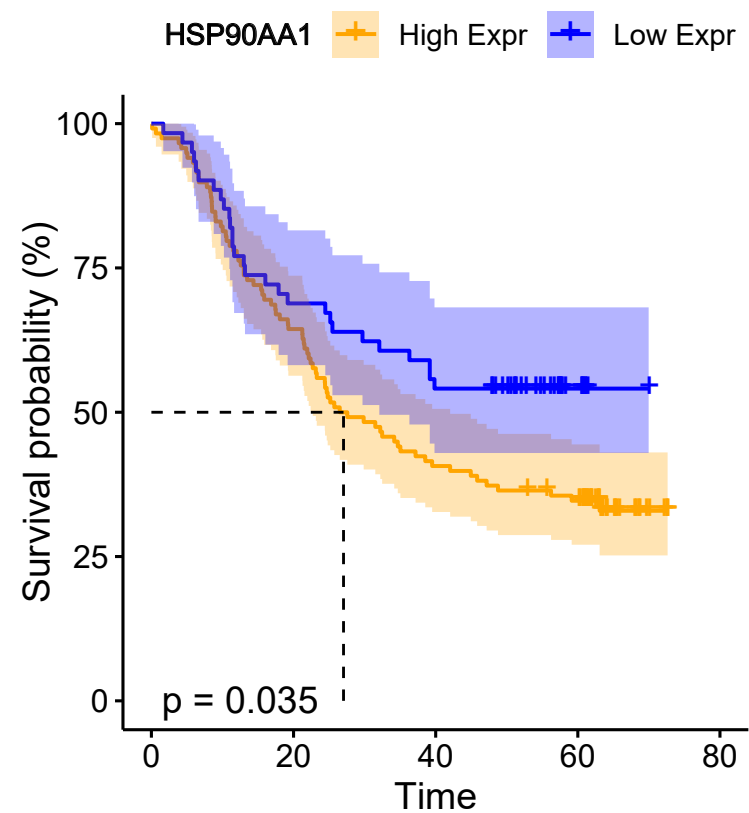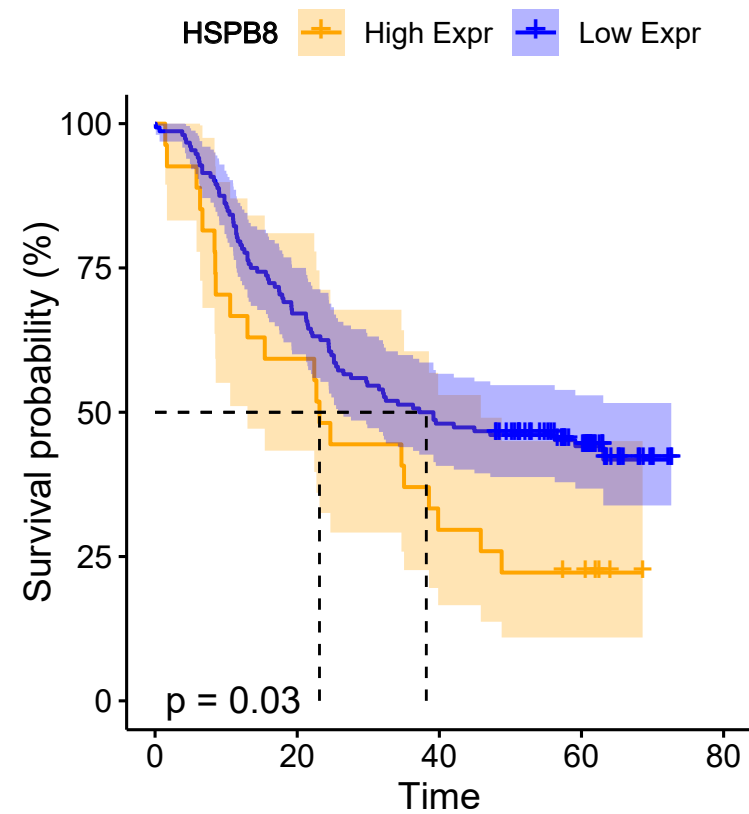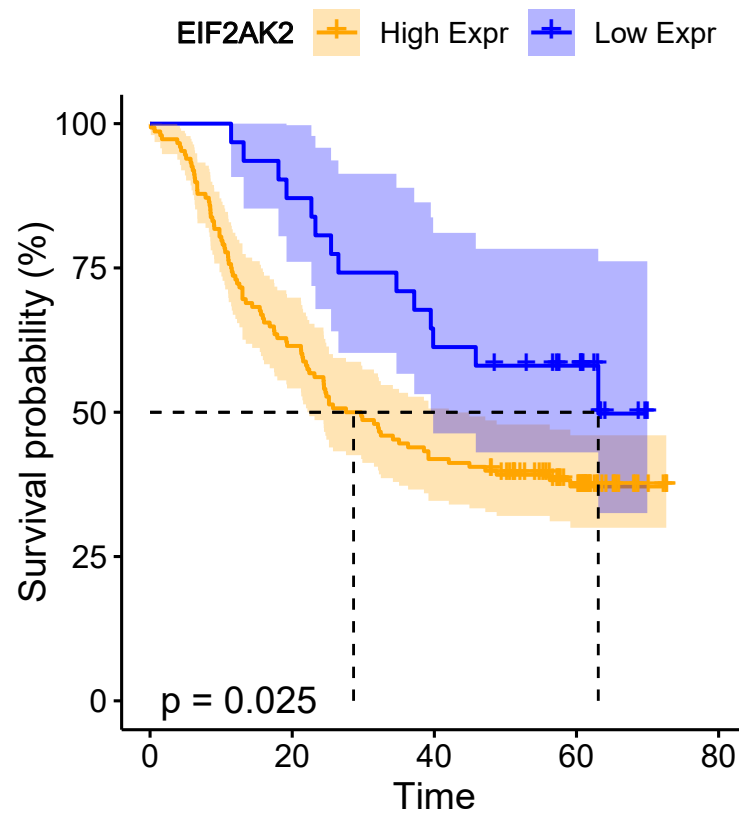

Supplement: Supplemental Information 3 [file peerj-12-17690-s003.pdf]
